# Exploring new avenues of health protection: plant-derived nanovesicles reshape microbial communities

**DOI:** 10.1186/s12951-024-02500-w

**Published:** 2024-05-19

**Authors:** Xiaohang Chen, Lianghang He, Chaochao Zhang, Genggeng Zheng, Shuoqi Lin, Yuchun Zou, Youguang Lu, Yan Feng, Dali Zheng

**Affiliations:** 1 Fujian Key Laboratory of Oral Diseases, School and Hospital of Stomatology, Fujian Medical University, Fuzhou, China; 2 Department of Preventive Dentistry, School and Hospital of Stomatology, Fujian Medical University, Fuzhou, China

**Keywords:** Plant-derived nanovesicles, Microbe, Crosstalk, Health care, Anti-infection

## Abstract

Symbiotic microbial communities are crucial for human health, and dysbiosis is associated with various diseases. Plant-derived nanovesicles (PDNVs) have a lipid bilayer structure and contain lipids, metabolites, proteins, and RNA. They offer unique advantages in regulating microbial community homeostasis and treating diseases related to dysbiosis compared to traditional drugs. On the one hand, lipids on PDNVs serve as the primary substances that mediate specific recognition and uptake by bacteria. On the other hand, due to the multifactorial nature of PDNVs, they have the potential to enhance growth and survival of beneficial bacterial while simultaneously reducing the pathogenicity of harmful bacteria. In addition, PDNVs have the capacity to modulate bacterial metabolism, thus facilitating the establishment of a harmonious microbial equilibrium and promoting stability within the microbiota. These remarkable attributes make PDNVs a promising therapeutic approach for various conditions, including periodontitis, inflammatory bowel disease, and skin infection diseases. However, challenges such as consistency, isolation methods, and storage need to be addressed before clinical application. This review aims to explore the value of PDNVs in regulating microbial community homeostasis and provide recommendations for their use as novel therapeutic agents for health protection.

**Graphical Abstract:**

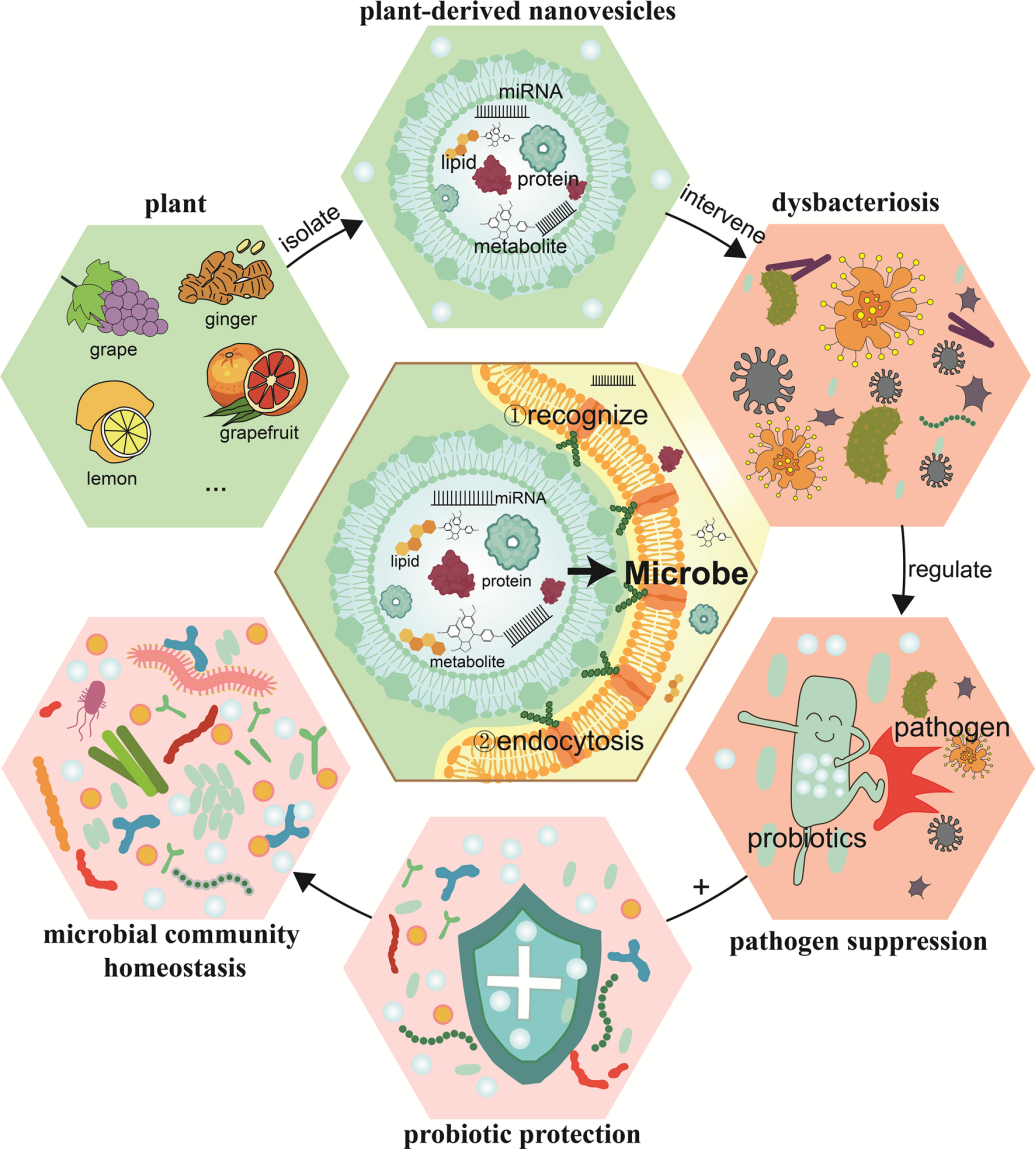

## Introduction

Maintaining a healthy and balanced microbiota is crucial for protecting human health [1]. Disruption of microbiota balance may lead to various inflammatory and infectious diseases [2]. Many factors are strongly associated with the maintenance or disruption of microbiota balance, among which dietary factors play an important role [3]. Dietary factors can influence the gut microbiota, thereby affecting human health. Fiber, compounds and proteins from plant sources are closely associated with the balance of the microbiota in the digestive tract [4]. In addition, more and more attention is beginning to focus on the role of plant-derived nanovesicles (PDNVs), especially those derived from fresh fruits and vegetables, in reshaping the microbiota balance [5].

PDNVs are distinguished from plant extracellular vesicles (PEVs) primarily based on the methods of preprocessing and isolation. PEVs are obtained through the infiltration of plant tissues or isolated from plant cell culture supernatants [6]. These PEVs play a crucial role in plants by delivering small RNAs (sRNAs) and messenger RNAs (mRNAs) to defend against external infections [7, 8]. Conversely, PDNVs are obtained by directly isolating plant tissues using differential centrifugation and density gradient centrifugation methods [9], which encompass not only extracellular vesicles but also other substances such as vesicle-like structure [6]. In this review, to avoid conceptual confusion, we focus specifically on the mechanisms, activities, and potential applications of PDNVs for human health protection, excluding PEVs. We focus on the cross-kingdom communication between PDNVs and microbiota to inspire applications in the field of biomedicine.

PDNVs are composed of lipid bilayer structures containing proteins, RNA, lipids and natural metabolites [10]. Variations in the degree of enrichment of their surface components determine their ability to be recognized and selectively taken up by different bacteria [11, 12]. It has been shown that the key to the specific uptake of PDNVs by microbiota lies in the differences in their surface lipid fractions [11]. These vesicles, enriched with various components, have the ability to simultaneously target multiple bacterial genes, including affect bacterial metabolic functions [13], modulate pathogenic gene expression in bacteria, reduce their pathogenicity [12, 14], and enhance the growth and survival of probiotics [11, 15]. These biological activities lay the foundation for PDNVs to regulate microbiota homeostasis. Therefore, PDNVs show great potential for application in the diseases such as periodontitis and colitis [12, 16].

Although the natural advantages of PDNVs have been extensively studied and the great potential of their cross-border communication with microbial communities has been recognized, there are still many aspects that need to be further summarized and thought about, including (1) how certain bacteria naturally recognize and endocytose PDNVs; (2) how PDNVs regulate gene expression for further regulating microbial bioactivities; (3) potential applications inspired by PDNVs with microbiota; and (4) key considerations for the clinical application of PDNVs. Therefore, this review aims to provide a comprehensive summary of the specific mechanisms, activities, and potential applications of cross-border communication between PDNVs and microbiota, and hopes to stimulate the prospects for the application of PDNVs as a new tool for regulating microbiota homeostasis and protecting human health.

## Overview: isolation and characterization of PDNVs

Previously, there has been increasing public interest in vesicle-like structures of plant origin. However, a notable issue that has caught attention is the inconsistency in nomenclature. Different designations have emerged in various studies, such as "extracellular vesicles" [17], "exosomes" [18], "extracellular vesicle-like nanoparticles" [19], "exosome-like nanoparticles" [15], "nanoparticles" [20], "nanovesicles" [21]. Given the differences in isolation methods, Pinedo et al., have called for a division into two categories [6].

The first category refers to vesicles naturally occurring in plants, collectively known as "plant-derived extracellular vesicles". These vesicles are obtained from supernatants derived from plant tissue cell culture [22] or by vacuum infiltration of plant tissues, primarily leaves [23]. The process involves tissue infiltration centrifugation to obtain an exosome wash, followed by purification through differential centrifugation, ultrahigh-speed centrifugation, and density gradient centrifugation. When exploring the physiological roles of extracellular vesicles in plants, such as their ability to resist external infections, researchers typically isolate plant-derived extracellular vesicles with higher purity [7, 8].

**Fig. 1:**
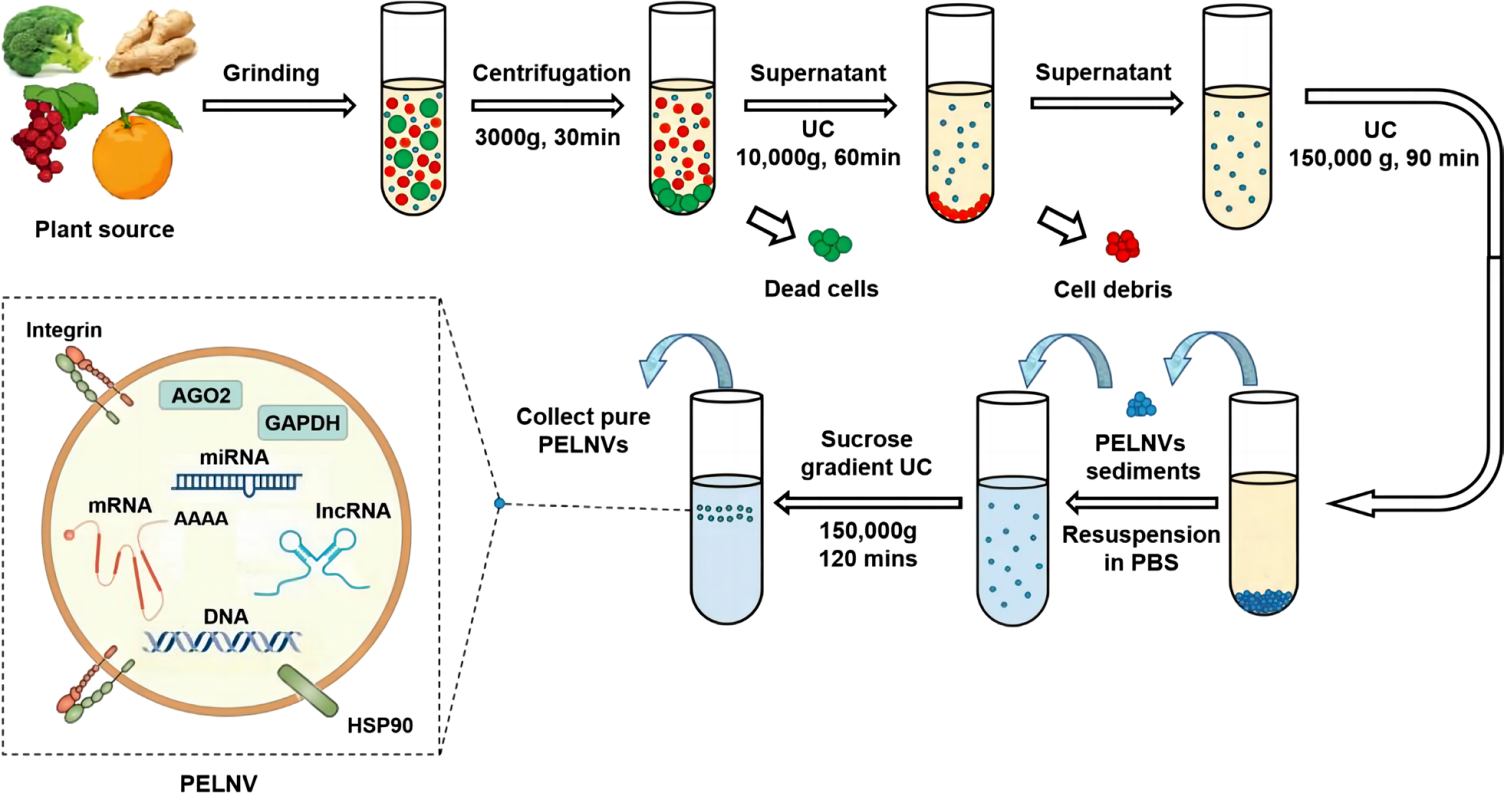
Preparation and isolation process of plant-derived nanovesicles. (*PELNVs* plant exosome-like nanovesicles, *UC* ultracentrifugation, *AGO2* Argonaute 2, *mRNA* messenger RNA, *PBS* phosphate-buffered saline, *GAPDH* glyceraldehyde-3-phosphate dehydrogenase.) (Copyright [26])

The second category comprises nanovesicles isolated through destructive processes from plant roots, leaves, and fruits. These nanovesicles are collectively referred to as "plant-derived nanovesicles". It is challenging to determine whether all these nanovesicles are naturally occurring extracellular vesicles. The isolation process involves grinding [24], extruding [25], or juicing [20], to directly obtain the juice, also followed by purification through differential centrifugation, ultracentrifugation, and density gradient centrifugation (Fig. 1). The key difference between the two categories lies in the pre-treatment step.

Direct tissue disruption in the isolation process leads to a higher presence of vesicle-like structures, including those that are not "extracellular vesicles" in the final product. This method allows for quicker and more substantial isolation of nanovesicles. For example, 48.5 ± 4.8 mg of nanovesicles could be isolated from 1 kg of ginger [27], and 18 ± 3 μg of nanovesicles could be obtained from 500 g of strawberry [28]. You et al. achieved an isolation yield of 1.504 × 10^11^ nanovesicle particles per gram of cabbage at a cost of approximately $0.00137, showcasing significant economic benefits [25]. This strategy has been predominantly used to explore the medicinal value or potential of PDNVs as drug delivery systems. Some studies have attempted to enhance yield or purity through electrophoresis [29], size exclusion [25], antibody capture [30], and polyethylene glycol (PEG) co-precipitation [22], although these methods are not mainstream. In this review, the focus is primarily on the regulation of microbial community by PDNVs as an inspiration for health protection. PEVs will not be extensively discussed to avoid confusion.

The characterization of PDNVs focused on their physical and biochemical features. Physical features mainly include morphology, potential, particle size and particle number. The morphology could be observed by techniques such as transmission electron microscopy or atomic force microscopy, and it mainly appears tea-to-shaped, with a stronger three-dimensionality under the microscope compared to solid nanoparticles. Potential, particle size distribution could be assessed using dynamic light scattering, nanoflow cytometry and nanoparticle tracking analysis. The potential of PDNVs is negative and the size usually ranges from 18 to 1000 nm [9], which is affected by centrifugation and purification methods as well as plant origin. Many studies have used nanoparticle tracking analysis or nanoflow cytometry to quantify the number of particles/mL in nanovesicles.

Biochemical characterization involves identifying the presence of RNA, proteins, lipids, and metabolites within the PDNVs. However, the current analysis of nanovesicle components is still far from complete. Researchers often use the bicinchoninic acid assay (BCA) to quantify protein concentrations in the nanovesicles. Some scholars also emphasize the importance of purity assessment, using indicators such as the number of particles/amount of protein (mg). Additionally, Triton X-100 could induce cleavage of membrane vesicle particles while having a minimal effect on non-membrane vesicle particles. Comparing the number of nanovesicles before and after cleavage could serve as an indicator to assess purity [31]. This is an issue that needs to be given high priority, as purity is an important guarantee of safety and an important parameter for translation into the clinic.

## Endocytosis: specific recognition and uptake by microbial communities

**Fig. 2:**
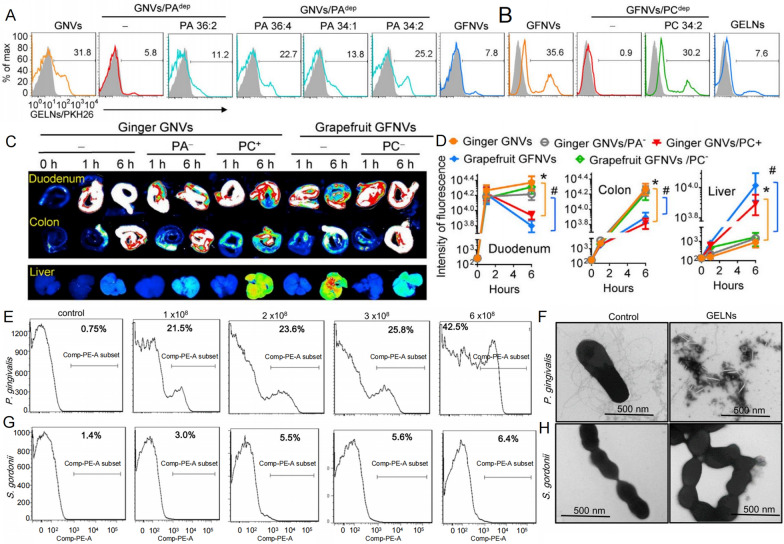
Differences in the uptake of plant-derived nanovesicles by bacteria. **A** Effect of different phosphatidic acid (PA) contents on the uptake efficiency of *Lactobacillus rhamnosus* *GG* during reconstitution of ginger-derived nanovesicles. **B** Effect of different phosphatidylcholine (PC) contents on the uptake efficiency of *Ruminococcaceae (TSD-27)* when nanovesicles of grapefruit origin are reconstituted. **C** Confocal laser scanning microscopy images of the contents of PC and PA in GNVs and GFNVs on their distribution in duodenum, colon and liver. **D** Statistical images of fluorescence intensity of PC and PA content in GNVs and GFNVs on their distribution in duodenum, colon and liver. **E** Uptake efficiency of *Porphyromonas gingivalis* by ginger-derived nanovesicles at different concentrations. **F** Effect of ginger-derived nanovesicles on the morphology of *Porphyromonas gingivalis*. **G** Uptake efficiency of ginger-derived nanovesicles at different concentrations against *Streptococcus gordonii*. **H** Effect of ginger-derived nanovesicles on the morphology of *Streptococcus gordonii*. (*GELNs* ginger-derived nanovesicles/ginger-derived exosomes like nanoparticles, *GNVs* GELN nanocarriers, *PA* phosphatidic acid, *PC* phosphatidylcholine, *GFNVs* grapefruit-derived ELN lipid nanocarriers, *P. gingivalis Porphyromonas gingivalis*, *S. gordonii Streptococcus gordonii*) (copyright [11, 12])

The surface of PDNVs has a variety of components, including substances such as lipids. Several studies have found that PDNVs are naturally targeted due to their enrichment with different substances on the surface [11, 12]. For example, researchers found that ginger- and turmeric- derived nanovesicles were predominantly taken up by *Lactobacillus rhamnosus GG* in mice. While grapefruit- and garlic-derived nanovesicles were predominantly taken up by *Ruminococcaceae (TSD-27)* [11]. By comparing the composition of nanovesicles from different plant sources, the researchers found that ginger and turmeric- derived nanovesicles were enriched in phosphatidic acid (PA) (35.2% and 34.4%, respectively), while grapefruit- and garlic- derived nanovesicles were enriched in phosphatidylcholine (PC) (36.2% and 52.6%, respectively). Further studies showed that PA was a key component of the nanovesicles targeting *Lactobacillus rhamnosus GG* (Fig. 2A), while PC was a key component of the nanovesicles targeting *Ruminococcaceae (TSD-27)* (Fig. 2B). In addition, these components were also associated with preferential targeting of the nanovesicles in vivo, with PA-rich nanovesicles tending to remain in the intestine, whereas PC-rich nanovesicles tended to migrate from the intestine to the liver. (Fig. 2C, D) [11].

This specific uptake phenomenon occurs not only in the gut microbial community, but also in the oral microbial community [12]. Researchers found that ginger-derived nanovesicles could be taken up by *Porphyromonas gingivalis* and inhibit its growth (Fig. 2E, F), but had less effect on *Streptococcus gordonii* (Fig. 2G, H) [12]. Ginger-derived nanovesicles are dose-dependently absorbed by *Porphyromonas gingivalis* but not by *Streptococcus gordonii* (Fig. 2E, G). By analyzing the composition of the ginger-derived nanovesicles, the researchers found that the lipid component played a key role in inhibiting *Porphyromonas gingivalis*. Further studies showed that 1-palmitoyl-2-linoleoyl-sn-glycero-3-phosphate (PA 34:2), the key composition of ginger-derived nanovesicles, inhibited the growth of *Porphyromonas gingivalis* at a very low concentration, whereas the inhibitory effect of ginger-derived nanovesicles on *Porphyromonas gingivalis* was diminished after removal of PA 34:2. In addition, the researchers found that the synthetic peptide of hemin-binding protein 35 (HBP35), a surface recognition of haemoglobin by *Porphyromonas gingivalis* that acts as the primary structure for pathogenesis, functional structural domain significantly inhibited the uptake of ginger-derived nanovesicles into specific microbial communities, suggesting that the interaction between nanovesicles and microbiota is based on the specific recognition of their surface components [12]. Overall, PDNVs interact with microbial communities through specific recognition and uptake by their surface components.

In addition, this was verified not only in ginger-derived nanovesicles, but also in tomato-derived nanovesicles. Gorevic et al. found that tomato-derived nanovesicles were also taken up by *Fusobacterium nucleatum* and that this uptake process was also dependent on lipid components [14]. When lipids were removed, the uptake of tomato-derived nanovesicles by *Fusobacterium nucleatum* was reduced and their growth state was restored [14]. In turn, nanovesicles reconstituted by cholesteryl esters (CE) were able to be taken up by *Fusobacterium nucleatum* [14]. These studies confirm the important role of lipids in the recognition of PDNVs by bacteria, but more studies are needed to confirm whether other components also play an important mediating role. Meanwhile, it paves the way for PDNVs as a natural drug-targeting delivery system. Qiao et al. have taken advantage of the property that ginger-derived nanovesicles are lipid-dependently taken up by bacteria, and loaded it as a carrier with Pb-Pt sheets that could be subjected to photo-therapy to kill bacteria more effectively in vivo [32].

Interestingly, the specific uptake phenomenon does not only occur in bacteria, but has also been studied at the cellular level. For example, tea leaves-derived nanovesicles with high expression of galactose moieties are more readily taken up by macrophages in the gut via galactose-mediated receptors [33]. In addition, β-glucan on the surface of oat-derived nanovesicles was able to target microglia cells in the brain [34], whereas lectin II on the surface of garlic-derived nanovesicles was able to target hepatocytes mediated by the cluster of differentiation 98 (CD98) receptor [35]. These experimental results reveal the potential natural targeting of PDNVs and provide a regular explanation about this phenomenon for further studies.

Research on the regulatory relationship between PDNVs and microbiota is in its infancy, and the specific recognition of PDNVs with microbiota that has been identified so far relies mainly on lipids, but because both microbial and plant species are so diverse, further research is needed to see whether this recognition involves other substances as well.

## Regulation: PDNVs synergistically regulate microbiota through multiple components

**Fig. 3:**
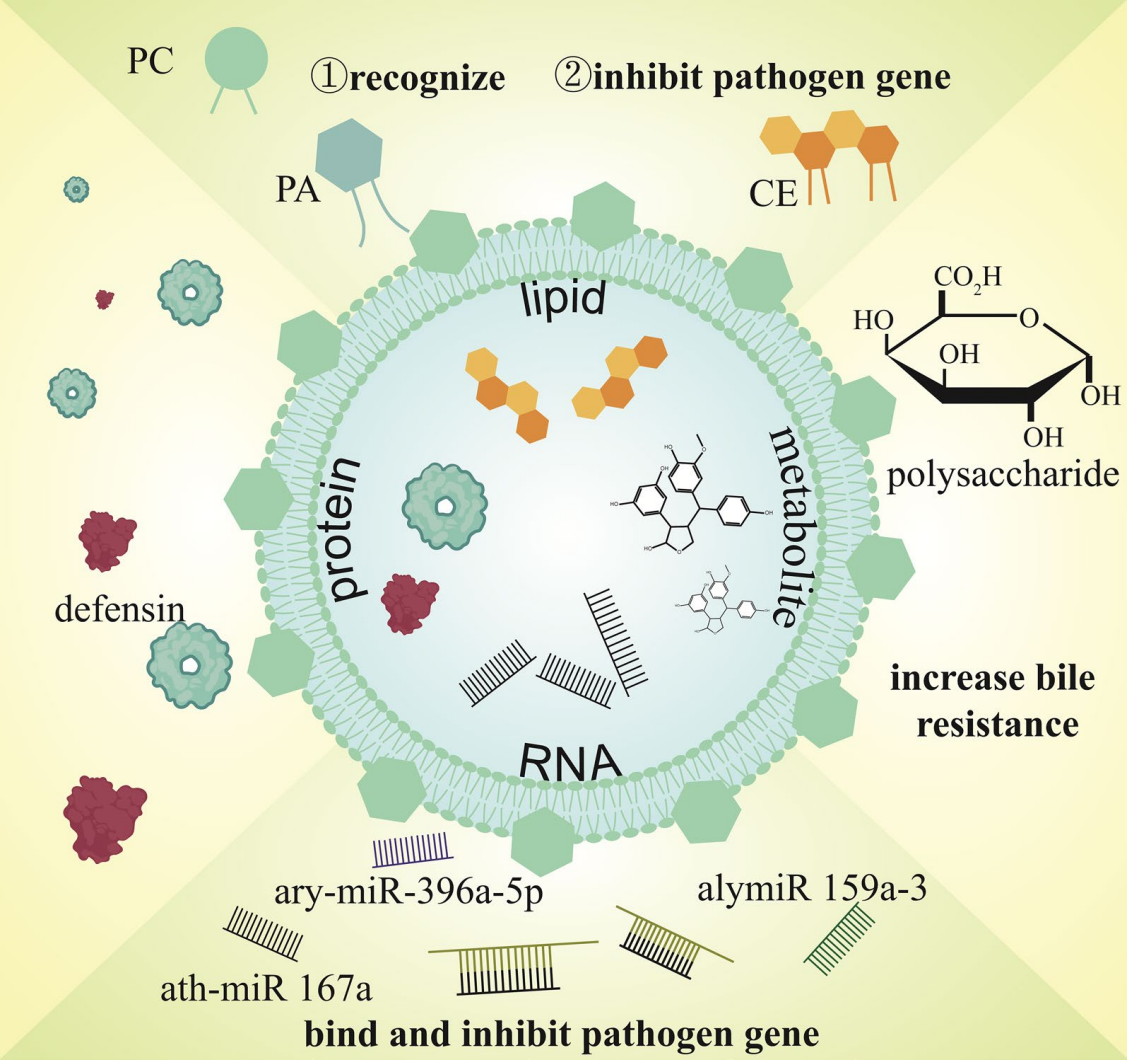
Different components of plant-derived nanovesicles regulating microbiota. (*PA* phosphatidic acid, *PC* phosphatidylcholine, *CE* cholesteryl esters)

PDNVs have been shown to contain multiple components such as lipids, metabolites, proteins and RNA. As a complex, these components in PDNVs often act synergistically to regulate microbiota (Table 1) (Fig. 3).

**Table 1:** Mechanisms of plant-derived nanovesicles in modulating microbiota

Plant	Isolation	Bioactivity	Animal model	Microbe	Mechanism	Refs.
Tartary buckwheat	UC	TBDNs significantly promoted *E. coli* and *LGG*, enhanced fecal microbial diversity, and increased short-chain fatty acid levels	Human	*E.coli* and *LGG*	A total of 129 microRNAs were identified by sequencing analyses, including 11 newly discovered microRNAs. Target gene prediction indicated that some of the microRNAs were able to target functional genes in physiological processes related to *E. coli* and *LGG*	[43]
Tomato	UC	Promoting the growth of probiotic *LGG* while inhibiting the growth of the conditional pathogens *C. difficile* and *F. nucleatum*	Smulator of intestinal microbial ecosystem model	*LGG*; *C. diff* and *F. nucleatum*	The antimicrobial effect of tomato-derived nanovesicles is driven by the presence of specific lipids in the nanovesicles, as demonstrated by lipid depletion and reconstitution experiments	[14]
Bee pollen	UC	Inhibition of *S. aureus* biofilm formation	–	*S. aureus*	–	[67]
Rehmannia	UC + D	Antimicrobial, inhibits biofilm formation	LPS-treated mice	*E.coli*	miR-7972 down-regulates the expression of GPR 161, activates the Hedgehog pathway, and inhibits biofilm formation in *E. coli* by targeting the virulence gene stx 2	[44]
Garlic	UC + D	Regulation of microbial community homeostasis	DSS-induced gut microbiota dysbiosis	–	–	[16]
Ginger	UC + D	Regulation of microbial community homeostasis	C57 BL/6 mouse/human	*LGG*	Ginger-derived nanovesicles is preferentially taken up by *LGG* in a lipid-dependent manner and contains microRNAs targeting various genes in *LGG*. Among these, ginger-derived nanovesicles mdo-miR7267-3p mediated targeting of the *LGG* monooxygenase ycnE produced increased I3 A. Ginger-derived nanovesicles-RNA or I3 A is sufficient to induce IL-22 production, which is associated with improved barrier function. These functions of ginger-derived nanovesicles-RNA can ameliorate colitis in mice through an IL-22-dependent mechanism	[11]
Ginger	UC + D	Elimination of exosome Nsp 12, Nsp 13-mediated induction of lung inflammation with ginger-derived nanovesicles aly-miR396a-5p	C57 BL/6 mouse	SARS-CoV-2	The role of ginger-derived nanovesicles in inhibiting SARS-CoV-2-induced cytopathic effect was further demonstrated by the inhibition of Nsp 12 and spike gene expression mediated by ginger-derived nanovesicles aly-miR396a-5p and rlcv-miRrL1-28-3p, respectively	[46]
Ginger	UC + D	Reduced pathogenicity of *P. gingivalis*, including inhibits *P. gingivalis* attachment to and invasion in oral epithelial cells	A mouse model of periodontitis	*P. gingivalis*	miRNAs and lipid ginger-derived nanovesicles are absorbed by *P. gingivalis* in a phosphatidic acid-dependent uptake by *P. gingivalis* and interact with haemoglobin-binding protein 35 on the P. *gingivalis* surface. Meanwhile, miRNAs and lipids of ginger-derived nanovesicles multi-targeted to inhibit virulence factors of *P. gingivalis*	[12]
Turmeric	UC + D	Regulation of microbial community homeostasis	DSS-induced gut microbiota dysbiosis	–	–	[40]
Tea flower	UC + D	Regulation of microbial community homeostasis	Mouse models of breast cancer	–	–	[37]
Tea leaf	UC + D	Regulation of microbial community homeostasis	IBD mice	–	–	[33]
Morus nigra L. leaves	UC + D	Regulation of microbial communityhomeostasis	HCC mouse model	–	–	[109]
Lemon	UC + D	Increase *LGG*’s bile resistance	In vitro gut microbiota culture	*LGG*	Lemon-derived nanovesicles limit the production of Msp1 and Msp3 by regulating the level of tRNA in *LGG* as a way to reduce the accessibility of bile to *LGG* cell membranes	[13]
Lemon	UC + D	Inhibit *C.diff*	*C. diff* infection (CDI) mouse model	*C. diff*, *LGG*, *STH*	Treatment with lemon-derived nanovesicles increased the levels of the AhR ligands I3 LA and I3 Ald, which induced the production of IL-22, and increased the lactate levels, which inhibited the growth and indole biosynthesis in *C. diff*. In addition, metabolites of *STH* inhibited the metabolic process of *LGG*, allowing fructose 1,6-bisphosphate (FBP) to accumulate in *LGG*. Subsequently, the accumulated FBP activated the lactate dehydrogenase of *LGG*, further enhancing the production of lactate and AhR ligand. A virtuous circle is formed	[15]

*UC* + *D* ultracentrifugation + density gradient centrifugation(sucrose), *TBDNs* Tartary Buckwheat derived nanovesicles, *LGG Lactobacillus rhamnosus GG*, *E. coli Escherichia coli*, *C. diff Clostridium difficile*, *F. nucleatum Fusobacterium nucleatum*, *S. aureus Staphylococcus aureus*, *LPS* Lipopolysaccharide, *GPR 161* G protein-coupled receptor 161, *stx 2* shiga toxin 2, *DSS* Dextran Sodium Sulfate, *I3 A* indole-3-carboxaldehyde, *IL-22* Interleukin-22, *Nsp 12* Nonstructural protein 12, *SARS-CoV-2* Severe Acute Respiratory Syndrome Coronavirus 2, *P. gingivalis Porphyromonas gingivalis*, *HCC* Hepatocellular Carcinoma, *Msp1* Mitochondrial Processing Peptidase Subunit 1, *STH Streptococcus thermophilus ST-21*, *CDI C. diff* infection, *I3 LA* indole-3-lactic acid, *I3 Ald* indole-3-carboxaldehyde, *AhR* Aryl hydrocarbon receptor

### Lipids

Lipids are a class of biomolecules that play a key role in membrane structure and function and play an important role in the regulation of microbiota with PDNVs [36]. Many studies have analyzed the lipid composition of PDNVs in their entirety and found significant differences in the lipid composition of nanovesicles from different plant sources. For example, the major lipid components in tea tree flower-derived nanovesicles were PC (about 26.6% of total lipids), triglycerides (TG, about 23.4% of total lipids), and phosphatidylethanolamine (PE, about 15.2% of total lipids) [37], whereas the major lipid components in tea leaves-derived nanovesicles were PA (about 32.7–45.7% of total lipids), phosphatidylglycerol (PG, 19.3–22.7% of total lipids), PC (13.6–19.2% of total lipids) and phosphatidylinositol (5.9–6.2% of total lipids) [11]. The different percentages of lipid components provide an important role for them to exert different biological effects.

Currently, researchers have found that the main roles of lipids from PDNVs in the regulation of microbiota fall into two aspects. Firstly, lipids in PDNVs play a role in mediating the cell-specific uptake of PDNVs [11], a very interesting role that has been explored in the previous section on endocytosis. Secondly, lipids in PDNVs play roles in exerting microbial regulation bioactivity. Sundaram et al. through further studies found that besides playing an important role in the binding and uptake process, PA 34:2 was found to inhibit *Porphyromonas gingivalis* growth at very low concentrations, and upon elimination of the PA 34:2, *Porphyromonas gingivalis*' growth capacity was restored [12]. PA (34:2) could inhibit the expression of multiple pathogenic gene mRNAs in *Porphyromonas gingivalis*. In addition, translocation of *Porphyromonas gingivalis* protease to the bacterial surface requires the type IX secretion system (T9 SS) [38]. Compared with the control, the treatment of ginger-derived nanovesicles total lipids significantly repressed the expression of 11 of the 12 T9 SS family genes. This suggests that the lipid components in PDNVs have antimicrobial activity and may regulate microbiota growth and metabolism by interfering with the expression of microbial pathogenic genes after mediating endocytosis.

### Proteins

PEVs have been shown to contain numerous proteins associated with defense functions [39]. However, proteins in PDNVs are still in the compositional analysis stage, with no related research exploring the specific mechanisms of these proteins' interaction with microbiota. Nevertheless, through further investigation into the roles of these proteins, we could gain a better understanding of the potential applications of PDNVs in the field of microbiology. For instance, Zhu et al. conducted proteomic analysis of garlic-derived nanovesicles and identified 61 proteins, including cytoplasmic proteins (β-actin), metabolic proteins (transferases), as well as membrane proteins such as lectins, aquaporins, and defense peptides [16]. The presence of these proteins suggests that PDNVs may play a significant role in the microbial defense response. Furthermore, Chen's study found a higher abundance of proteins in tea tree flower-derived nanovesicles, with up to 745 species identified, including transmembrane proteins and cytoplasmic proteins, all exceeding 15 kilodaltons in molecular weight [37]. The presence of these proteins could influence the metabolism and growth processes of microbiota, further impacting their pathogenicity and ecological functions. Turmeric-derived nanovesicles have protein molecular weight ranging from 4 to 26 kilodaltons [40]. According to KEGG enrichment analysis, approximately 70% of the proteins in turmeric-derived nanovesicles play a crucial role in regulating microbiota [40]. The content of these proteins may be related to the method of isolation, with different layered nanovesicles isolated using the density gradient method containing different amounts of proteins. Liu et al., found that turmeric-derived nanovesicles contained no proteins in one layer, while the other layer was rich in proteins [41]. These proteins' specific functions may involve antimicrobial, anti-inflammatory, and microbiota regulation abilities. Through in-depth research into the mechanisms of action of these proteins, we could uncover the interplay between PDNVs and microbiota, providing more specific insights for biomedical applications.

### RNA

The RNA components of PDNVs have unique functions in microbial regulation, especially the microRNAs (miRNAs) have been widely studied in inhibiting microbial activities. miRNAs lead to degradation or inhibition of translation of bacterial mRNAs by complementary pairing with bacterial mRNA sequences [42]. Zhang's team investigated the role of miRNAs in nanovesicles of various plant origins on gut flora remodeling. They found that ginger-derived nanovesicles contained 109 miRNAs. Some of the miRNAs could be detected in the faeces of mice after the administration of these PDNVs, indicating the stability of these miRNAs. In addition, some of these miRNAs could inhibit the invasion of *Lactobacillus rhamnosus GG* strains into intestinal epithelial cells, such as ath-miR 167a which directly binds to the mRNA of the *Lactobacillus rhamnosus GG* bacterial hair protein streptococcal surface protein C (SpaC) and regulates the expression of SpaC, which significantly reduces the metastasis of *Lactobacillus rhamnosus GG* strains to the peripheral blood and retains *Lactobacillus rhamnosus GG* strains on the surface of the intestinal mucosa [11]. These regulations are very friendly to the intestinal microenvironment.

Similarly, Liu et al. isolated tartary buckwheat-derived nanovesicles, which were found to contain 129 miRNAs. They identified miR 6300, miR 482 b, miR 482c, novel 1, and miR 3630 as some of the target miRNAs. By gene ontology (GO) enrichment analysis, they found that these miRNAs may target functional genes in *Escherichia coli* and *Lactobacillus rhamnosus GG*, including the regulation of physiological processes such as cell cycle, DNA replication, organic matter transport and glucose metabolism in bacteria. By synthesizing mimics of these miRNAs, they found that the miR 6300 mimic could promote the growth of *Escherichia coli* [43]. Conversely, Qiu et al. found that 12 miRNAs were present in Remannia-derived nanovesicles, among which miR-7972 had the highest abundance. They found that miR-7972 could bind to the bacterial virulence gene shiga toxin 2 (stx2), thereby inhibiting the growth of *Escherichia coli* [44]. These studies show that different PDNVs exert apparently different effects due to differences in their components.

Sundaram et al. resolved the key role of PDNVs in regulating the pathogenic genes of *Porphyromonas gingivalis*. In ginger-derived nanovesicles, miRNAs could synergize with lipids to inhibit the pathogenic activity of *Porphyromonas gingivalis*. Many miRNAs could bind to pathogenic gene loci, for example, alymiR 159a-3 could bind phage-associated protein A (phagA) and arabinose operon regulatory protein C (araC), while gma-166p also has a potential binding site in outer membrane protein A (ompA). These miRNAs regulate mRNA expression of arabinose operon regulatory protein C (AraC), hemagglutinin A (HagA), outer membrane protein A (OmpA), and rod-shaped protein A (RodA), thereby decreasing lysine-specific gingipain (Kgp) and arginine-specific gingipain (RgpA and RgpB) activities [12]. These examples of miRNAs and lipids synergistically regulating pathogenic genes in pathogenic bacteria highlight the unique advantage of naturally enriched multicomponents in PDNVs.

These PDNVs not only have a regulatory effect on bacterial pathogenicity, but also have an inhibitory effect on viruses. One study used a computer simulation approach for target prediction and found that miRNAs in certain PDNVs could specifically target severe acute respiratory syndrome coronavirus 2 (SARS-CoV-2) but not SARS-CoV [45]. Another study found by validation that ginger-derived nanovesicles may contain 135 miRNAs capable of binding and inhibiting mRNA expression on SARS-CoV-2. The researchers also found that mimics of lcv-miR-rL1-28-3p and ary-miR-396a-5p were expected to significantly reduce S and non-structural protein 12 (Nsp 12) expression levels in SARS-CoV-2 [46]. It is important to note that many studies have used miRNA mimics in downstream experiments, which do not fully mimic the real concentration of miRNAs in PDNVs. In addition, since PDNVs are complexes, the concentration of miRNAs in them cannot be well modelled.

Overall, miRNAs in PDNVs play an important role in microbial regulation. They affect bacterial growth and metabolism by pairing with bacterial RNA sequences. These findings reveal the unique functions of PDNVs miRNAs and provide valuable clues for further research into the use of these miRNAs for microbial regulation and therapy. It is worth noting that RNA components other than miRNAs may also be present in PDNVs, especially in the case of PEVs, where it was found that extracellular vesicles secreted by Arabidopsis thaliana cells could deliver sRNAs [7] and mRNAs [8] to *Staphylococcus*, silencing disease-causing genes that play a key role or translationally expressing them in the causative organisms in order to reduce the infection of the plant. As such, there is much to explore in this area.

### Metabolites

Regarding the metabolites in PDNVs, current studies have also mainly focused on compositional analysis, and relatively few studies have been conducted on their regulatory effects on microbial community. However, as one of the main components of PDNVs, the role played by metabolic compounds in regulating the microbiota cannot be ignored.

There have been studies indicating differences in metabolic compound compositions in PDNVs, some of which have shown antimicrobial activity. For example, green tea contains major catechin compounds, namely epigallocatechin gallate (EGCG) and epicatechin gallate (ECG), which have been extensively researched and found to possess antibacterial properties [47]. These compounds have been observed to inhibit a wide range of bacteria, including both Gram-positive and Gram-negative strains. Tea tree flowers- and tea leaves-derived nanovesicles are abundant in flavonoids and polyphenolic compounds, including EGCG and ECG [33, 37]. Curcumin, also a metabolite found in PDNVs, demonstrates inhibitory effects against various bacteria, including Gram-positive bacteria like *Staphylococcus aureus* and *Streptococcus aureus*, and Gram-negative bacteria such as *Escherichia coli* and *Salmonella* [48]. It exerts its antimicrobial effects through several mechanisms, including disruption of bacterial cell membrane integrity, inhibition of bacterial biofilm formation, interference with bacterial biosynthetic pathways, and inhibition of bacterial growth and replication [49].

Interestingly, a study on lemon-derived nanovesicles has confirmed the important role of metabolites in PDNVs in the regulation of microbiota. Lei et al. found that lemon-derived nanovesicles contained a galacturonic acid-enriched pectin-type polysaccharide, which significantly increased the bile resistance of *Lactobacillus rhamnosus GG*. This was achieved by decreasing the levels of tRNA_ser_^UCC^ and tRNA_ser_^UCG^ of *Lactobacillus rhamnosus GG*. Consequently, the secretion of proteins such as mitochondrial processing peptidase subunit 1 (Msp 1) and Msp 2 from *Lactobacillus rhamnosus GG* was reduced. This finding is consistent with the decay of tRNA mediated by RNase P [13]. These studies demonstrated the important role of components in PDNVs in regulating gene expression and metabolic processes in microbial community (Table 1).

## Bioactivity: microbial regulation by PDNVs

PDNVs are active when they are selectively taken up by microbe and enter the microbe to regulate their genes. Currently, many studies have demonstrated that many PDNVs could protect probiotics, fight against harmful bacteria, and alter the bacterial metabolism, which in turn could regulate the microbial community, especially intestinal microbe.

### Protecting probiotics

Probiotics play an important role in microbial community imbalance [50]. They compete with harmful bacteria for resources and space, inhibit their excessive proliferation and maintain the balance of the microbial community [51]. Probiotics produce beneficial metabolites, such as short-chain fatty acids, which regulate intestinal function and immune response, and promote the health of the intestinal mucosa [52]. In addition, they participate in digestion and nutrient absorption, modulate immune system function, and enhance host resistance [53]. However, microbiota imbalance may lead to dysbiosis, affecting the number and diversity of probiotics and causing diseases. A number of studies have been conducted to show that the intake of PDNVs favours the growth of probiotics, as well as protects them from extreme environments such as bile, helping to promote gut health and overall well-being [11].

*Lactobacillus rhamnosus GG* is an intestinal probiotic that is important for controlling intestinal microbe, establishing a bioprotective barrier in the gut, and enhancing immunity [54]. It has been found that *Lactobacillus rhamnosus GG* in intestinal tissues selectively endocytosis ginger-derived nanovesicles contain a variety of miRNAs. This uptake increases the amount of *Lactobacillus rhamnosus GG* while potentially altering other bacterial metabolites. [11]. Compared to treatment with no nanovesicles, when *Lactobacillus rhamnosus GG* was intervened with miRNA from ginger-derived nanovesicles, the level of inflammation in intestinal tissues was reduced, and the production of interleukin-22 (IL-22) was increased. Ginger-derived nanovesicles also modulated the transcript and protein levels of *Lactobacillus rhamnosus GG* and inhibited the invasion of intestinal mucosal cells by harmful bacteria for intestinal protection. In addition, tomato-derived nanovesicles could also promote the growth of *Lactobacillus rhamnosus GG* [14, 15].

When probiotics enter the body, viability is often compromised due to extreme microenvironments such as bile and stomach acid. PDNVs provide a protective strategy. The lemon-derived nanovesicles enhanced the resistance of *Lactobacillus rhamnosus GG* to bile and increased their survival. The interaction of probiotics with lemon-derived nanovesicles also altered the composition of intestinal metabolites, increased lactic acid and indole production, decreased the number of *Clostridium difficile*, and prolonged the presence of probiotics in the gut [15]. Taken together, PDNVs could exert their activities through interactions with probiotics, including increasing the number of probiotics, modulating intestinal inflammation, and protecting intestinal tissue. These findings suggest that PDNVs may play an important role in protecting intestinal health.

### Fighting opportunistic pathogenic bacteria

Opportunistic pathogenic bacteria have an unfavourable effect when the microbial community is out of balance [55]. The imbalanced state of the organism provides opportunities for these bacteria to grow and multiply, enabling them to resist competition and control [56]. Toxins and virulence factors produced by opportunistic pathogens cause damage to host tissues and immune systems, increasing the risk of infection and disease [57]. The maintenance of a balanced microbial community is essential and the adverse effects of opportunistic pathogens could be prevented and controlled by promoting the growth, diversity and healthy lifestyle of beneficial bacteria [58]. Appropriate use of antibiotics, adequate nutrition and strengthening of immune system function are also key [59]. Maintaining a balanced microbial community reduces the risk of infection and disease, thus helping to protect human health. However, certain pathogens such as *Clostridium difficile *[60], *Fusobacterium nucleatum* [61], *Porphyromonas gingivalis* [62], *Staphylococcus aureus* [63], and *Escherichia coli* [64] are common and can disrupt the microbial community, posing a threat to human health. Recent studies have shown that PDNVs may be a potential tool to inhibit the growth and activity of these pathogenic bacteria.

Both *Clostridium difficile* and *Fusobacterium nucleatum* are common intestinal pathogens [65]. Infections caused by *Clostridium difficile* are usually associated with antibiotic misuse and their main symptoms are diarrhoea and abdominal pain. *Fusobacterium nucleatum* is also an intestinal pathogen associated with food poisoning and gastrointestinal infections [66]. Lee et al. found that tomato-derived nanovesicles could have an inhibitory effect on the growth of both *Clostridium difficile* and *Fusobacterium nucleatum* through growth curve experiments [14]. Similarly, *Porphyromonas gingivalis* is one of the most important causative agents of oral lesions and is closely related to periodontal diseases. Sundaram et al. found that ginger-derived nanovesicles not only inhibited the growth of *Porphyromonas gingivalis*, but also reduced the adhesion and invasion of *Porphyromonas gingivalis* to oral epithelial cells [12].

*Staphylococcus aureus* is also a highly pathogenic bacterium that mainly produces a variety of virulence factors, such as surface proteins, exotoxins, and various enzymes that cause local and even systemic tissue inflammation [63]. Bee pollen-derived nanovesicles were found to have an inhibitory effect on the ability of *Staphylococcus aureus* to form biofilms. This is important for reducing the severity of *Staphylococcus aureus* infections, as biofilm formation makes the bacteria more difficult to remove and increases their resistance to antibiotics [67]. In addition, dandelion-derived nanovesicles have been found to neutralize its virulence factors for inhibiting the hemolytic reaction caused by *Staphylococcus aureus* [68]. *Escherichia coli* is a common intestinal bacterium that could also cause severe food poisoning and infections [69]. miR-7972 enriched in Remannia-derived nanovesicles may exert an inhibitory effect on the expression of the virulence gene Stx2 *caused by Escherichia coli*. This not only reduced the growth of *Escherichia coli* but also inhibited its biofilm formation [44]. Reduced biofilm formation is important for the effective application of antibiotics, as antibiotics become less effective against biofilms. Therefore, the application of PDNVs may also potentially increase bacterial susceptibility to drugs such as antibiotics. All these studies support the favorable role of PDNVs in the inhibition of opportunistic pathogenic bacteria.

### Alteration of bacterial metabolism

**Fig. 4:**
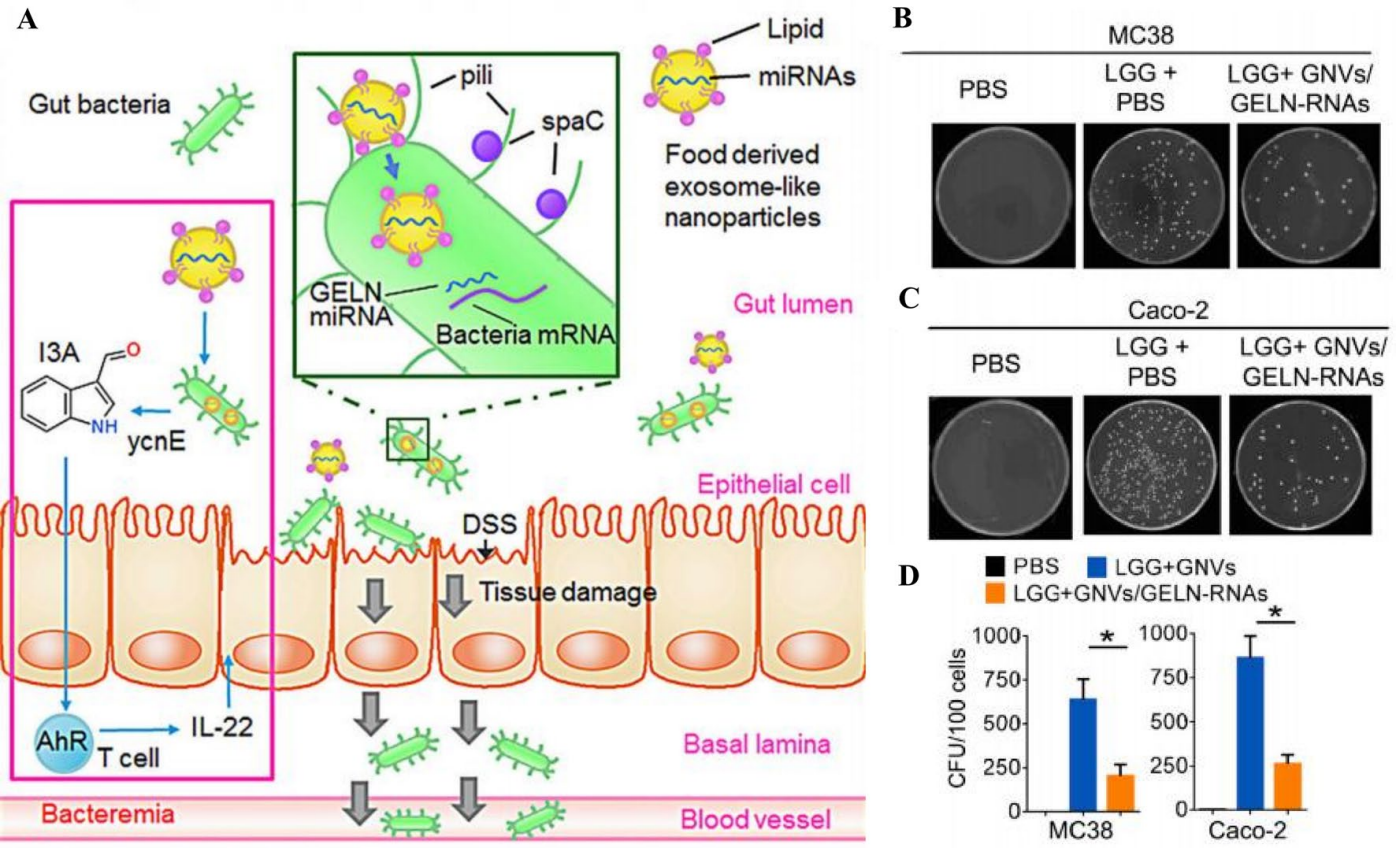
PDNVs derived miRNAs in intestinal flora remodeling. **A** PDNVs are ingested by intestinal bacteria and produce more I3A in the presence of ycnE, which binds to AhR and produces IL-22 in the presence of T cells to protect the intestinal barrier. **B**, **C**, **D** Cloning of *LGG* co-cultured with phosphate buffered solution (PBS) or GNVs/GELN-RNAs and statistical analysis (*P < 0.05). (Copyright [14]) (*I3A* Indole-3-carboxaldehyde, *ycnE* yeast conserved nucleolar protein E, *AhR* Aryl Hydrocarbon Receptor, *IL-22* Interleukin-22, *LGG Lactobacillus rhamnosus GG*, *PBS* Phosphate buffered solution, *GELNs*, ginger-derived nanovesicles, *GNVs* GELN nanocarriers)

The metabolic activity of microbiota has a significant impact on bacterial pathogenicity and organismal health [70]. Some microbes produce beneficial metabolites through metabolism, such as short-chain fatty acids, which can promote gut health and immune system function [53]. The series of effects played by altering the metabolism of bacteria is also one of the main activities of PDNVs in regulating the microbial community [71]. A study found that lemon-derived nanovesicles enhanced the level of lactate production by lactic acid bacteria, thereby inhibiting the growth of *Clostridium difficile*. This was achieved by increasing the production of the aryl hydrocarbon receptor (AhR) ligands indole-3-lactic acid (I3 LA) and indole-3-carboxaldehyde (I3 Ald), leading to the induction of IL-22 and the accumulation of lactate, which inhibited *Clostridium difficile* growth and indole biosynthesis (Fig. 4A) [15]. Compared to single PBS treatment, ginger-derived nanovesicles decrease the clone formation of *Lactobacillus rhamnosus GG* (Fig. 4B, C). Also, Qiu et al. found that Remannia-derived nanovesicles might enhance glycerolipid metabolism, cytoskeletal proteins, β-alanine metabolism and lipid metabolism in bacteria and inhibit nitrogen metabolism, folate biosynthesis, and cancer pathways in the gut microbiota [44]. But these may be based on data analyses that need more data to confirm.

When dietary fiber is fermented by probiotics in the gut, it produces a range of short-chain fatty acids [53]. Short-chain fatty acids are a class of organic acids consisting of two to six carbon atoms, of which the three most common are acetic acid, propionic acid and butyric acid [72]. These short-chain fatty acids could be used as a source of nutrients to supply bacteria with the ability to lower intestinal pH, maintain proper acidity and alkalinity, inhibit the growth of harmful bacteria, and act as an immunomodulator. It has been found that different doses of garlic-derived nanovesicles treatment could alter the composition of short-chain fatty acids produced by the gut microbiota. In particular, 100 mg/kg garlic-derived nanovesicles treatment led to a significant increase in the concentration of acetic acid, butyric acid, propionic acid, valeric acid, isobutyric acid and isovaleric acid [16]. This is important for the improvement of intestinal diseases such as colitis. Tartary buckwheat-derived nanovesicles also significantly increased the content of short-chain fatty acids during faecal fermentation [43]. This finding suggests that PDNVs may act as a potential nutrient factor to promote nutrient absorption and utilization by gut microbes, thereby modulating gut health.

### Regulation of overall microbial community homeostasis

The balance of the microbial community is crucial for maintaining the health, and imbalances could lead to diseases [73]. Within the microbial community, both pathogenic and probiotic bacteria play significant roles [74]. The proliferation of pathogenic bacteria, such as *Salmonella spp.* and *Escherichia coli*, especially when they become dominant, could lead to intestinal infections, resulting in symptoms such as diarrhea, vomiting, and abdominal pain [75]. These bacteria damage the intestinal mucosa and immune system by producing toxins or invading host tissues. On the other hand, probiotics like *Lactobacillus* and *Bifidobacterium* have a positive impact on organismal health [76]. They promote food digestion and nutrient absorption, enhance intestinal barrier function, inhibit the growth of pathogenic bacteria, and produce beneficial metabolites like short-chain fatty acids [77]. These metabolites help regulate immune system function and reduce inflammatory responses. Several studies have shown that the administration of PDNVs leads to an increase in the abundance and diversity of the intestinal microbe. This increase is mainly due to a higher proportion of probiotic bacteria and a decrease in opportunistic pathogenic bacteria (Table 2).

**Table 2:** Modulation of intestinal microbiota by plant-derived nanovesicles

		Tomato	Turmeric	Garlic	Tea leaves	Rehmannia	Tartary buckwheat	Tea tree flower	Morus nigra L. leaves	Ginger
Probiotics	Akkermansia	↑	↑	↓	↑					
	Lactobacillus	↑	↑			↑				
	Firmicute			↑	↑	↑		↑	↓	
	Bacteroidetes				↑	↓		↑	↓	↑
	Oscillibacter				↓					
	Clostridiaceae									↓
	Lactobacillus	↑	↑			↑	↓		↑	
	Lysinibacillus						↑			
	Anoxbacillus						↑			
	Ruminococcaceae				↑					↓
	Staphylococcus		↓							
	Lachnospiraceae			↑	↑					
	Clostridiaceae									↓
	Megasphaera						↓			
	Leuconostoc						↑			
	Bifidobacterium		↑		↑					
	Parabacteroides	↑								
	Prevotella	↓				↑			↓	
	Fusobacterium						↓			
	Norank Muribaculaceae								↓	
	Turicibacter								↑	
	Patescibacteria								↑	
Conditional pathogen	Klebsiella						↑			
	Escherichia		↓	↓	↓		↑		↓	
	Enterobacter						↓			
	Citrobacter						↓			
	Serrati						↑			
	Pseudomonas aeruginosa						↓			
	Proteobacteria			↓	↓					
	Helicobacter		↓	↓	↓					
	Salmonella						↑			
	Salmonella typhimurium						↓			
	Shigella		↓	↓		↓			↓	
	Vibrio						↓			
	Desulfovibrio				↓					
	Brachyspira				↓					
Refs.		[14]	[40]	[16]	[33]	[44]	[43]	[37]	[109]	[11]

The regulation of gut microbial community homeostasis by PDNVs has been validated in various models, including gastrointestinal microbial simulation systems, dextran sodium sulfate (DSS)-induced colitis models [16] in mice or *Clostridium difficile*-infected mice [15], mouse tumor models, and even human volunteers [11]. For example, Lee et al. used fermentation simulators for gut microbes and found that tomatoes-derived nanovesicles could promote the proliferation of *Lactobacillus*. while inhibiting the growth of *Clostridium spp*. [14]. Liu et al. conducted clinical trials on the modulation of intestinal microbiota by Tartary buckwheat-derived nanovesicles. They observed an increased diversity of the intestinal microbiota and found that the nanovesicles fine-tuned the microbial community without significantly affecting probiotic proliferation [43]. Higher flora diversity is beneficial for the balance of intestinal flora, regulation of the immune system and intestinal nutrient absorption. Turmeric and tea leaves-derived nanovesicles up-regulated important probiotic like *Bifidobacterium*, which are crucial for protecting intestinal health [33, 40, 78]. *Bacteroidetes*, a phylum that aids in cellulose degradation, nutrient absorption, and metabolism, were up-regulated by tea [33], ginger [11], and tea tree flower-nanovesicles [37]. On the other hand, turmeric [40], garlic [16], and tea leaves-derived nanovesicles [33] down-regulated the abundance of the conditionally pathogenic bacterium *Helicobacter*, including *Helicobacter pylori*, a leading cause of chronic gastritis and peptic ulcers.

While not every PDNVs showed the same trend for specific probiotics or pathogenic bacteria, overall, many PDNVs increased the diversity of the intestinal microbe. This indicates the potential of PDNVs as orally administered drugs with good biocompatibility, paving the way for further research in this area.

## Application potential: PDNVs regulate microbial community for health protection

**Fig. 5:**
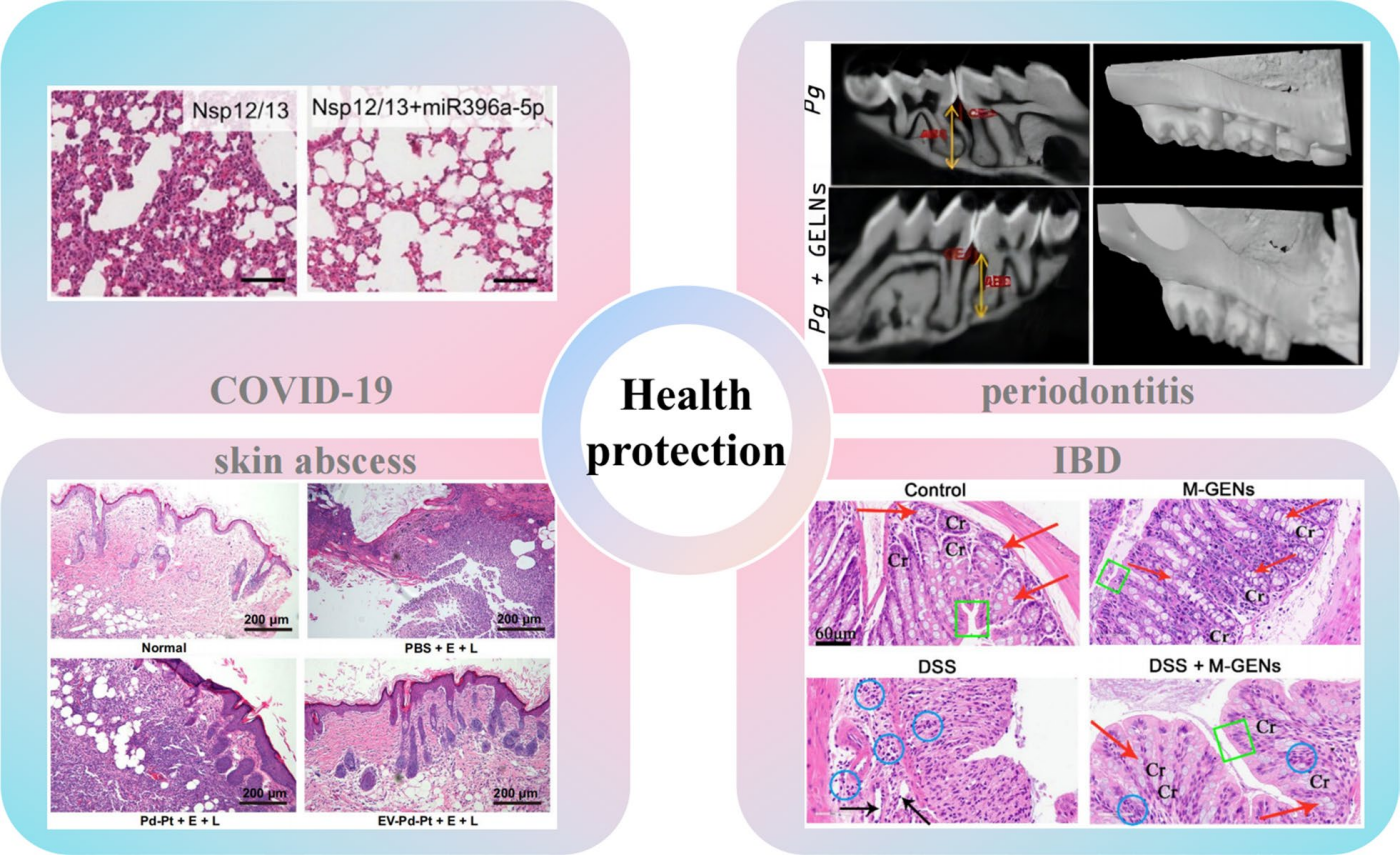
Plant-derived nanovesicles protect tissue health through microbial regulation. (*COVID-19* Coronavirus Disease 2019, *IBD* inflammatory bowel disease, *Pg* Porphyromonas gingivalis, *GELNs* ginger-derived nanovesicles, *EV-Pb-Pt* + *E* + *L* ginger-derived nanovesicles modified with Pb-Pt slices + electric field + photothermal, *DSS* dextran sodium sulfate, *GENs* garlic-derived nanovesicles, *M* medium.) (copyright [12, 16, 32, 46])

From the above summary of the effects of PDNVs on microbiota, it could be found that PDNVs have a powerful effect on regulating the microbial community homeostasis, especially on the intestinal microbe, which has even been verified in human trials. As a major "organ" of the human body, intestinal microbial community has been shown to be closely related to many diseases [79], and PDNVs have been shown to have therapeutic potential in periodontitis [12], inflammatory bowel disease [16], skin infection [32], COVID-19 [46] and other diseases(Fig. 5). The potential applications as probiotic protectants and for the synergistic treatment of other diseases by modulating the homeostasis of the organism's microbial community are also in the spotlight [15]. These studies suggest the great potential of PDNVs in protecting the health.

### Treatment of periodontitis

Periodontitis is an infectious disease that affects 20–50% of the world's population and not only leads to tooth loss but is also associated with systemic diseases including diabetes [80]. Current treatments for periodontal disease focus on mechanical removal of plaque to further control the condition, followed by the use of antibiotic controlled-release gels [81]. *Porphyromonas gingivalis* is the main causative agent in periodontal disease and could lead to alveolar bone loss [82]. Compared with conventional treatments, PDNVs are expected to play a multi-target modulation role in periodontal disease treatment, reducing the emergence of drug resistance while being biosafety. Ginger-derived nanovesicles are dose-dependently absorbed by *Porphyromonas gingivalis* and carry lipids and miRNAs that act synergistically to bind to multiple pathogenic gene targets, thereby reducing their virulence. Sundaram et al. showed that ginger-derived nanovesicles in drinking water for 10 consecutive days significantly reduced the proportion of *Porphyromonas gingivalis* in the oral cavity of mice and reduced its ability to colonize the oral cavity [12]. In vitro experiments confirmed that the ability of *Porphyromonas gingivalis* to invade and adhere to oral keratin epithelial cells was reduced in the presence of ginger-derived nanovesicles. In an animal model of periodontitis, resorption of alveolar bone and infiltration of pro-inflammatory factors and inflammatory cells, including leukocytes and macrophages, were significantly reduced. These results suggest that ginger-derived nanovesicles could remodel the oral microbiota and act as guardians of oral health [12].

### Treatment of inflammatory bowel disease

Inflammatory bowel diseases (IBD), including ulcerative colitis (UC) and Crohn's disease, are chronic diseases characterized by inflammation of the digestive tract [83]. Due to their increasing prevalence, these diseases have become a major health concern worldwide. The etiology of IBD remains elusive due to a combination of genetic predisposition, environmental triggers, and aberrant immune responses [84]. Current treatments focus on suppressing the immune response, but sometimes with side effects [85]. This highlights the need to explore alternative therapeutic strategies. The mechanism of PDNVs for the treatment of inflammatory bowel disease has two main aspects. On the one hand, they act as anti-inflammatory agents and antioxidants in IBD, which could provide a favourable environment for local tissue regeneration [86]. On the other hand, they remodel the dysregulated gut microbiota. The two complement each other and ultimately have a therapeutic effect on IBD and could prevent the development of cancer.

The pathology of IBD is the aberrant activation of the toll-like receptor 4 (TLR4) signalling pathway [87]. Overstimulation triggers a cascade of inflammatory responses that damage the intestinal mucosa. Both garlic and mulberry bark-derived nanovesicles have shown the ability to counteract this overactivation [16, 88]. Garlic-derived nanovesicles contain specific miRNAs, such as Han-miR3630-5p, that bind to the 3' untranslated region of TLR4 and inhibit its expression, which in turn downregulates the expression of myeloid differentiation primary response gene 88 (MyD88) and nuclear factor kappa-B (NF-κB). This leads to a reduction in pro-inflammatory cytokines, thereby alleviating intestinal inflammation [16]. In addition, the aryl hydrocarbon receptor (AHR) also plays a key role in IBD [89]. Activation of AHR could regulate the function of immune cells and inhibit the inflammatory response and activation of immune cells. Heat shock protein family A (HSP70) member 8 (HSPA8) in mulberry bark-derived nanovesicles binds to the AHR and selectively activates constitutive photomorphogenesis 9 (COP9)/COPS8 in the intestinal epithelium, thereby protecting the intestinal epithelium, which is essential for the prevention of DSS-induced colitis in mice [88].

The intestinal barrier also plays a key role in preventing pathogen penetration. In IBD, this barrier is disrupted [90]. Turmeric-derived nanovesicles were shown to preserve the integrity of the intestinal barrier due to the reduction of e-calmodulin, zonula occludens-1 (ZO-1), and occludin, which subsequently modulates ulcerative colitis. This effect may be related to the fact that turmeric-derived nanovesicles could modulate the phosphoinositide 3-kinase/ protein kinase B (PI3K/Akt) signalling pathway, cytokine receptor interactions, cell adhesion molecules and NF-κB signalling pathways [40]. And the garlic and mulberry bark-derived nanovesicles also showed the potential to strengthen this barrier. By promoting the expression of tight junction proteins, they reduce the risk of bacterial translocation and thus reduce inflammation [16, 88].

The large increase in IL-22 levels in colonic mucus is a marker of the potential impact of ginger-derived nanovesicles on intestinal tissue protection, which depends on the AHR pathway for its synthesis and also requires the participation of *Lactobacillus rhamnosus GG*. In response to the activity of the monooxygenase yeast conserved nucleolar protein E (ycnE), mdo-miR7267-3p in GELNs facilitates the targeted increase in indole-3-carboxaldehyde (I3A) production by *Lactobacillus rhamnosus GG*. I3A (a ligand for the aromatic hydrocarbon receptor) is sufficient to activate IL-22 production, which is linked to better barrier function. It's also interesting to note that *Lactobacillus rhamnosus GG*'s SpaC gene encourages the bacterium's in vivo migration to other organs, whereas ginger-derived nanovesicles contained ath-miR167a-5p reduces SpaC transcript and protein levels, dramatically inhibiting *Lactobacillus rhamnosus GG* invasion of intestinal mucosal cells [14]. All of these studies demonstrate the great promise of the application of PDNVs as an oral agent for the maintenance of intestinal health homeostasis. Clinical trials using ginger-derived nanovesicles as curcumin carriers to assess efficacy and biosafety for treating IBD, but the results have not been published (Table 3).

**Table 3:** Clinical trials of plant-derived nanovesicles (from www.clinicaltrials.gov)

Plant	Conditions	Aim	Sample	Interventions	Administration	Indicator	Status	Identifier
Plant	Colon cancer	To investigate the ability of plant exosomes to deliver curcumin more efficiently to normal colonic tissues and colonic tumors	35 participants	A: Binding of curcumin to plant exosomes B: Curcumin C: No intervention	Administered orally for 7 consecutive days	Serum cytokine levels; safety and tolerability; effects on metabolic profiles of normal colonic mucosa and colonic tumors; immunohistochemistry of cells in vitro	Recruiting	NCT01294072
Ginger	Irritable bowel disease (IBD)	Edible structures within plant cells (ginger) have clinically important anti-inflammatory effects on the intestinal lining of patients with IBD	90 patients with chronic IBD	1. ginger exosomes; 2. curcumin; 3. ginger exosomes plus curcumin	Ginger exosome plus curcumin was administered orally once daily for 28 days	Sigmoidoscopy and biopsy, blood tests, quality of life questionnaire	Completed	NCT04879810
Grape	Head and neck cancer; oral mucositis	To assess the ability of grape exosomes given to subjects to act as an important anti-inflammatory agent in reducing the incidence of oral mucositis during radiation and chemotherapy treatment of head and neck tumors	60 participants	Experimental: 1-Grape extract; 2-Lortab, fentanyl patch, and mouthwash daily during radiotherapy and chemotherapy treatment	Administered orally for 35 consecutive days	Pain caused by oral mucositis; level of immune biomarkers in blood and mucosal tissue	Completed	NCT01668849

### Treatment of skin infection

The multiple advantages of PDNVs, including mass production potential, ease of accessibility, natural palatability, environmental sustainability, and favourable biosafety, make them a promising adjuvant strategy [10, 91]. The combined application of PDNVs with other nanomaterials could greatly amplify the therapeutic efficacy. Nanomaterials offer a variety of unique advantages and are particularly promising in the field of antimicrobials [92]. For example, certain nanomaterials could disrupt plaque biofilms by triggering chemical reactions under acidic pH conditions specific to plaque biofilms, which could impede bacterial growth by generating reactive oxygen species through their own enzyme-like activity [93]. The physicochemical properties of nanomaterials could also produce enhanced antimicrobial effects, such as photothermal and photocatalytic effects, which are triggered by near infrared radiation (NIR) light or electric fields [94]. Nonetheless, as there have been concerns about the stability of nanomaterials in the bloodstream and so on, PDNVs could precisely compensate for these regrets [95].

**Fig. 6:**
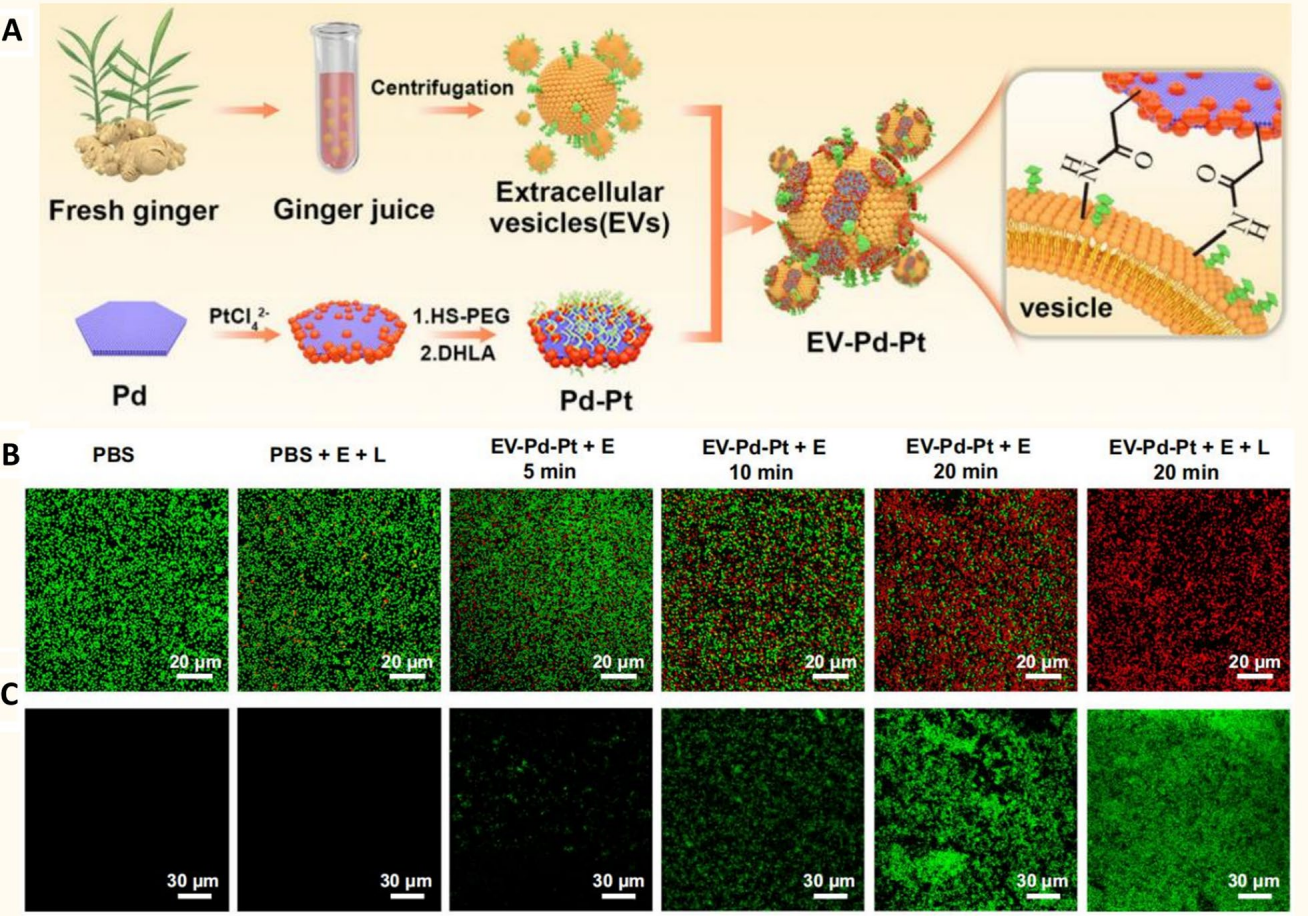
Potential applications of engineered PDNVs in antibacterial field. **A** Pb-Pt was activated and attached to ginger-derived nanovesicles. Carboxyl groups on the surface of Pb-Pt and amino groups on the surface of extracellular vesicles formed amide bonds. **B** Fluorescence images of bacterial live-dead under different interventions. (Green fluorescence represents live bacteria and red fluorescence represents dead bacteria.) **C** Fluorescence images of bacterial reactive oxygen species production under different interventions. (Green fluorescence represents bacteria labelled by the DCFA fluorescent probe, which is primarily used to detect reactive oxygen species levels.) (NIR = 980 nm) (*PDNVs* plant-derived nanovesicles, *E* electric field, *L* light, *NIR* near-infrared light, *DCFA* Dichlorofluorescein.) (Copyright [32])

Pb-Pt has electrocatalytic and photothermal properties [96]. By activating Pb-Pt, its surface hydroxyl groups could form amide bonds with the amino groups on the surface of ginger-derived nanovesicles, which leads to the successful attachment of Pb-Pt to ginger-derived nanovesicles (Fig. 6A). Based on the fact that the ginger-derived nanovesicles could be taken up by bacteria lipid-dependently, Pb-Pt could be synergistically absorbed. Then, in the presence of NIR and an electric field, Pb-Pt undergoes a hydrolysis reaction that generates a large amount of reactive oxygen species, thus killing the bacteria (Fig. 6B, C) [32]. This strategy was confirmed in a rat abscess model, where the biomimetic ginger-derived nanovesicles could prolong the half-life of Pb-Pt, interact with bacteria more effectively, and induce an antibacterial effect. In vivo infection modelling have shown that the infection was almost negligible in the intervention of this ginger-derived nanovesicles@ Pb-Pt, and in vitro bacterial cultures of the infected portion also confirmed bacterial clearance [32]. This is an example of PDNVs coupled with nanomaterials to exert anti-infective effects, and based on the many excellent properties of PDNVs, it is believed that more sparks will collide with other nanomaterials.In wound infections, in addition to direct bacterial inhibition, there is a need to inhibit toxins released by bacteria, such as the multiple exotoxins contained in the extracellular vesicles released by *Staphylococcus aureus* [97]. These exotoxins promote bacterial colonization and lead to impairment of the immune response. By interfering with the action of these exotoxins, bacterial resistance to drugs could be reduced. Since PDNVs are rich in multiple substances, they can regulate multiple genes in bacteria, which has greater therapeutic potential than a single target. Tan et al. also found that dandelion-derived nanovesicles were able to specifically bind to exotoxins in the extracellular vesicles of *Staphylococcus aureus*, preventing the hemolytic reaction that they triggered [68]. They loaded these dandelion-derived nanovesicles into light-curable methacryloyl-modified gelatin, which prolonged their retention time and concentration at the wound site and better penetrated the skin tissue. This bionic hydrogel loaded with dandelion-derived nanovesicles demonstrated the ability to combat bacterial infection, reduce inflammation, promote cell migration, and accelerate chronic wound healing in an infected wound model. These strategies bridge the therapeutic effects of PDNVs with the strengths and weaknesses of the materials field, with a large translational potential.

### Treatment of COVID-19

Coronavirus disease 2019 (COVID-19) is an infectious disease caused by a novel coronavirus that poses a great challenge to global public health [98]. It faces many bottlenecks in diagnosis and treatment, especially therapy, and some therapeutic potentials have been inspired from PDNVs [99]. Through computer simulations, researchers predicted 11 miRNAs in PDNVs that could bind SARS-CoV-2 [45]. Furthermore, Teng et al. used experiments to validate the treatment of ali-miR 396a-5p in ginger-derived nanovesicles blocked exosomal Nsp 12/Nsp 13-mediated NF-κB activation, which subsequently prevented apoptosis and excessive inflammatory responses in lung epithelial cells. Intratracheal delivery of ali-miR 396a-5p in ginger-derived nanovesicles inhibited lung inflammation induced due to viral Nsp 12, while suppressing SRAS-CoV-2 cytopathic effects in vero E6 cells (kidney cell line) by inhibiting viral S and Nsp 12 expression [46]. This study shows that PDNVs, which contain multiple components, not only naturally act on viral genes, but also modulate excessive cellular immune responses, avoiding "cytokine storms" and other damages to the body, which is a significant advantage for health protection.

### Probiotic synergists

Probiotics have a huge market potential due to their potential benefits to human health and wide range of applications [100]. However, the probiotics market faces a number of issues, including the issue of survivability of probiotics once they enter the body [101]. Studies have shown that PDNVs are expected to play an important role in the successful functioning of *Lactobacillus rhamnosus GG* into the body. On the one hand, lemon-derived nanovesicles could limit the production of Msp 1 and Msp 3 by *Lactobacillus rhamnosus GG*, thereby reducing bile damage to the cell membrane. Among them, Ribonuclease P (RNase P), an essential housekeeping nucleic acid endonuclease, is responsible for lemon-derived nanovesicles-induced decay of tRNA_ser_^UCC^ and subsequently regulates the downregulation of Msp levels. Whereas, galacturonic acid-rich pectin-type polysaccharides are active factors in lemon-derived nanovesicles that increase bile resistance and downregulate tRNA_ser_^UCC^ levels in *Lactobacillus rhamnosus GG *[13]. On the other hand, it has been shown that the combination of lemon-derived nanovesicles and 11 probiotics could reduce the mortality rate of *Clostridioides difficile* infection to 40%. The addition of *Streptococcus thermophilus ST-21* (STH) and *Lactobacillus rhamnosus LR-32* (LRH) to these 11 probiotics further reduced the mortality rate from 40 to 20%. Whereas, lemon-derived nanovesicles play an important role in protecting probiotics against bile resistance. Lemon-derived nanovesicles with STH and LRH treatment increased the AhR ligands indole-3-lactic acid (I3 LA) and indole-3-carboxaldehyde (I3 Ald) leading to induction of IL-22 and increased lactic acid leading to the inhibition of *Clostridioides difficile* by inhibiting its growth and indole biosynthesis [15]. This protection and synergistic exertion of effects is of great potential for application, suggesting that PDNVs could not only serve as nutrients to nourish probiotics, but also protect them.

### Potential synergistic modulation of other diseases

There is an association between gut microbiota and non-intestinal diseases, including systemic diseases and other organ pathologies [102]. It has been found that dysbiosis or deficiency of gut microbiota may be associated with the development and progression of systemic diseases such as obesity, diabetes, and cardiovascular disease [103]. In addition, abnormal changes in intestinal microbiota are also closely associated with other organ pathologies such as IBD [104], autoimmune disease [105] and neurological disease [106], as well as the development of tumors [107]. While exploring PDNVs as disease therapeutic agents, many studies have found that these PDNVs could not only have a direct therapeutic effect on the relevant diseases, but also found that these vesicles have a regulatory effect on the intestinal microbiota. This is understandable, because by gavage or oral administration, the PDNVs entering the intestinal tract do not reach the lesion site completely from the intestinal tract, and most of them "react" with cells and microbes in the gastrointestinal tract. At the time of disease, the gastrointestinal function is weakened, and the modulation of the intestinal microbiota by these PDNVs is expected to strengthen the body's resistance and pave the way for the elimination of the disease [16, 88].

For example, microbe have been shown to be significantly associated with the development of tumors such as metastasis and proliferation [108]. Chen et al. isolated tea flower-derived nanovesicles and found that in addition to the direct modulation of proliferation, apoptosis and mobility in cancer cells, they also had a significant effect on the maintenance of the stability of the intestinal microbiota after oral administration by decreasing the abundance of typically harmful bacteria and increasing the percentage of probiotic bacteria [37], especially the *Firmicutes* mentioned above [40]. And in liver tumours, Gao et al. found that oral administration of mulberry leaf-derived nanovesicles increased the relative abundance of *Lactobacillus* and *Turicibacter*, and that the increased *Lactobacillus* may perhaps attenuate galactose-induced hepatic injury in rats by inhibiting hepatic inflammation, enhancing the intestinal barrier function, and modulating the regulated metabolome of the gut microbiota [109]. Of course, this phenomenon has been found not only in tumourigenesis and development, but similar findings have been made in liver injury [44]. However, the current study is only at the stage of phenomenon discovery, and there is no direct correlation study, which deserves further investigation.

## Key considerations: the challenge of stability and reproducibility of PDNVs

**Fig. 7:**
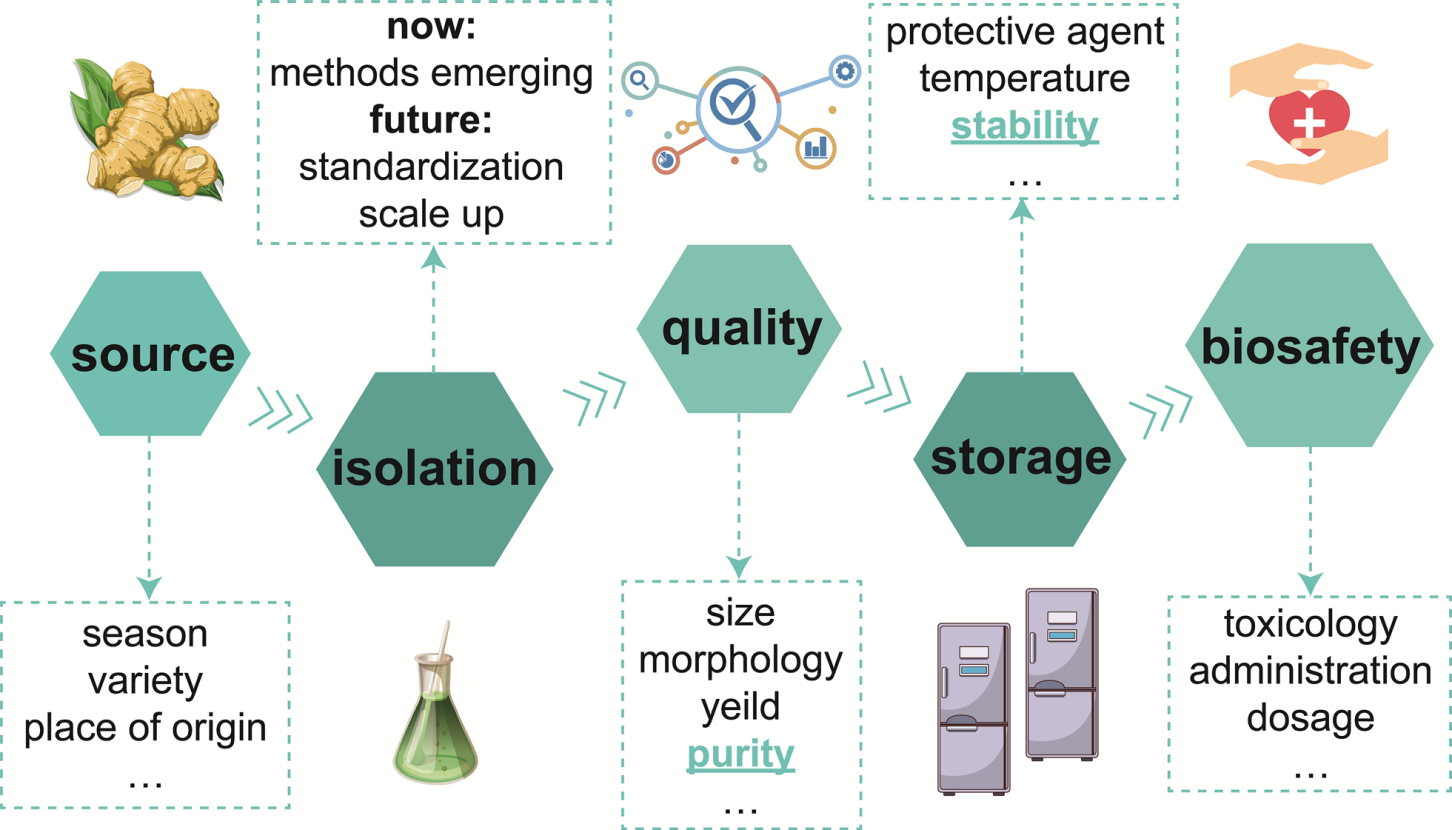
The key consideration of PDNVs for clinical application

As mentioned above, PDNVs, when taken up by microbes, can modulate microbial community homeostasis and have great potential for health protection. However, there are many challenges that need to be brought to the forefront before PDNVs go to the clinic (Fig. 7). PDNVs are widely available and are a green and renewable resource. But there wide range of sources poses a challenge for the consistency of the composition of PDNVs. Plants are subject to a wide range of interfering factors during growth, including climate, water, light, nutrients, seasons, and so on, all of which could affect plant components [110]. Although there are few laws about the entry of plant sorting substances into the nanovesicles, the contents of the nanovesicles are conceivably bound to change with the components of the plant [10]. Therefore, it is important to assess the batch reproducibility as well as normalization of PDNVs, which may have differences in contents. And each time histology or sequencing is still relatively expensive, so the development of convenient methods for batch validation of PDNVs is also one of the important directions in the future.

In addition, while the source could be controlled, a standardized process should be established for the isolation of PDNVs. At present, the main bottleneck lies in two aspects, on the one hand, the isolation scheme about PDNVs is still in a stage of competition to find a better way for the time being. The currently considered gold standard, the use of density gradient method combined with differential centrifugation, suffers from low yield, and because PDNVs are separated mainly on the basis of their density, this method also fails to remove pigments and heteroproteins of similar density [25, 111]. And the acquisition method with high purity and high yield still requires extensive experiments. On the other hand, it currently relies mainly on morphological observations based on transmission electron microscopy, particle size and potential distribution under dynamic light scattering, nanoparticle tracking analysis, and nanoflow cytometry, as well as simple identification of its contents. The plant sources are too wide to have a similar key marker that could be used as an indicator for the identification of PDNVs similar to the one used for extracellular vesicles in mammalian-derived cells. It has been suggested that penetration 1 (PEN1), the ATP-binding cassette transporters (ABC) transporter, and penetration 3 (PEN3) (usually involved in fungi penetration resistance) and the Tetraspanin-8 (TET8), among others, are promising markers for PEVs, but there is not yet a have a relevant consensus [6]. Therefore, further research on PDNVs is expected to establish standards for the isolation and identification of PDNVs earlier. Fortunately, more and more groups and experts have reached some consensus on the study of PDNVs [6, 31].

After obtaining PDNVs, there are challenges to the contents of PDNVs such as seasonal variability, which poses a huge challenge to the efficacy of PDNVs. At this point, it is important to consider how to preserve PDNVs. Existing preservation of PDNVs is mainly done by simply lowering the temperature, with most of the PDNVs being stored at −80 °C, some of the PDNVs undergo liquid nitrogen flash freezing and then stored at −80 °C, and there are also studies of storing them at −20 °C or place them at 4 °C for short-term preservation. There have been some studies evaluating the storage of PDNVs at different temperatures for short periods of time, and they found that 1 month's storage at some temperatures caused these PDNVs to undergo morphological changes, mainly in the form of aggregation or fusion [112]. Of course, this is related to the origin of the plant, some PDNVs are relatively more stable, and different PDNVs may be suitable for storage at different temperatures [112]. Some studies have shown that the effect of transdermal treatment of melanoma with aloe vera-derived nanovesicles as a delivery vehicle for indocyanine green (ICG) remains after storage for some time [113]. However, systematic evaluation is lacking. In addition, there are studies that used the preservative 1,3-butylene glycol (1,3-BG) or Trimethylolethane(TMO) for the preservation of leaf-derived nanovesicles from plants and found that preservation at 4 °C showed higher stability under the protection of TMO, but this study only evaluated preservation temperatures of −20 °C, 4 °C, 25 °C and 45 °C [114]. These studies suggest that further studies are needed to evaluate the suitable preservation conditions for PDNVs, and even to develop suitable lyophilized protective agents, which is an important part of moving PDNVs towards the clinic.

The biosafety of PDNVs for humans is the most critical aspect. Although many of the PDNVs studied so far are food-borne, such as tomato, ginger, lemon, and garlic, these components are present in our natural foods. In addition, most of the studies have also used administration methods such as oral or topical administration, which are not prone to elicit significant immune responses. However, when these nanovesicles are consumed with the pulp, the effects on the human body may not be consistent with those produced when high concentrations of nanovesicles are ingested. Therefore, there is an urgent need for a method to visualize the metabolic pathways by which nanovesicles from plants such as natural fruits enter the body. In addition, herbal plant-derived nanovesicles also account for a significant proportion of PDNVs, and whether these herbal plant-derived nanovesicles are immunogenic or potentially biotoxic needs to be further considered, as herbal medicines contain substances such as alkaloids that may be potentially toxic [31]. Although many studies have demonstrated the good biocompatibility of PDNVs, including the absence of organ inflammation, the absence of elevated hepatotoxicity and nephrotoxicity indices, as well as the absence of crossing the placental barrier [115], more studies are needed to demonstrate the activity and safety of PDNVs in the human body.

Many in vivo distribution experiments have confirmed that PDNVs, despite being non-homologous to the animal body, could remain in the body for a long period of time and are not cleared by the immune system in a transient manner [116]. However, it is important to note that this clearance is affected by the mode of administration, and studies have shown that oral administration of tea tree flower-derived nanovesicles has a better biosafety profile, whereas intravenous administration activates the C3 complement system and also leads to weight loss in mice [37]. At the same time, very detailed pharmacotoxicological testing of PDNVs is not yet available, and the results of pharmacotoxicological testing may vary from plant to plant, so there is still much room for improvement. Evidence from clinical trials of PDNVs is ongoing (Table 3) (https://clinicaltrials.gov/), but there are no relevant data omissions from completed clinical trials either, and results regarding Phase I clinical trials have not yet been reported. Therefore, more evidence specific to PDNVs in humans is needed to demonstrate their activity and safety in vivo before PDNVs can be used as therapeutic agents or drug delivery vehicles. Therefore, there are still many critical issues waiting for further breakthroughs.

## Conclusion

In conclusion, diet is closely related to the organism's microbiota balance, and various foods consumed on a daily basis contain varying amounts of nanovesicles, a lipid bilayer structure composed of lipids, proteins, RNAs, and metabolites whose cross-border communication with microbes is expected to inspire novel therapeutic modalities. The lipids in these PDNVs are the main tools to mediate bacterial uptake and further regulate genes and metabolism in microbes through their contents. Based on this, PDNVs exhibit numerous activities in reshaping microbiota homeostasis, including reducing the pathogenicity of harmful bacteria, protecting probiotics, and subsequently acting as a regulator of microbial community homeostasis. Due to these activities, PDNVs have great potential in the treatment of periodontitis, inflammatory bowel disease, and other diseases associated with microbial dysbiosis. However, before entering the clinic, PDNVs should be evaluated and explored in all aspects of plant source batch reproducibility, standardization of isolation methods and characterization, exploration of preservation methods, and biosafety comprehensive assessment. Despite the many problems, at least three clinical trials on PDNVs are underway. This small but ample PDNVs are expected to play a great role in protecting human health in the future.

## Data Availability

Data sharing is not applicable to this article as no datasets were generated or analyzed during the current study.
